# A high throughput imaging database of toxicological effects of nanomaterials tested on HepaRG cells

**DOI:** 10.1038/s41597-019-0053-2

**Published:** 2019-05-02

**Authors:** Elisabeth Joossens, Peter Macko, Taina Palosaari, Kirsten Gerloff, Isaac Ojea-Jiménez, Douglas Gilliland, Jaroslav Novak, Salvador Fortaner Torrent, Jean-Michel Gineste, Isabella Römer, Sophie Marie Briffa, Eugenia Valsami-Jones, Iseult Lynch, Maurice Whelan

**Affiliations:** 1 European Commission, Joint Research Centre (JRC), Ispra, Italy; 2 School of Geography, Earth and Environmental Sciences, University of Birmingham, Edgbaston, Birmingham UK; 3 Present Address: Human & Environmental Health & Safety Group, Materials Safety Unit, LEITAT, C/Palllars 179-185, 08005 Barcelona, Spain

**Keywords:** Nanotoxicology, Cellular imaging

## Abstract

The large amount of existing nanomaterials demands rapid and reliable methods for testing their potential toxicological effect on human health, preferably by means of relevant *in vitro* techniques in order to reduce testing on animals. Combining high throughput workflows with automated high content imaging techniques allows deriving much more information from cell-based assays than the typical readouts (i.e. one measurement per well) with optical plate-readers. We present here a dataset including data based on a maximum of 14 different read outs (including viable cell count, cell membrane permeability, apoptotic cell death, mitochondrial membrane potential and steatosis) of the human hepatoma HepaRG cell line treated with a large set of nanomaterials, coatings and supernatants at different concentrations. The database, given its size, can be utilized in the development of *in silico* hazard assessment and prediction tools or can be combined with toxicity results from other *in vitro* test systems.

## Abstract (summary)

**Table:** 

Design Type(s)	compound treatment design • factorial design
Measurement Type(s)	toxicity
Technology Type(s)	high content screening
Factor Type(s)	concentration • coating • nanomaterial
Sample Characteristic(s)	Homo sapiens • Hepa-RG cell

Machine-accessible metadata file describing the reported data (ISA-Tab format)

## Background & Summary

The increasing use of manufactured nanomaterials (MNMs) in food, industrial processes and consumer products poses a significant challenge to industry and regulatory bodies with regard to demonstrating their safety^1–4^. Issues being faced include the often peculiar properties exhibited by MNMs which can lead to unpredictable behaviour in biological systems and the growing pressure to move away from traditional animal tests used for toxicological hazard assessment due both to ethical and scientific reasons. There is strong interest therefore to pursue the development and application of *in vitro* and *in silico* methods which have the potential to progressively replace animal testing for the generation of toxicological data that can support the safety assessment of MNMs in a variety of contexts^5^.

The combination of *in vitro* High Throughput Screening (HTS) and High Content Imaging (HCI) delivers rapid and reliable toxicity assessment of large numbers of MNMs in parallel and can combine several endpoint measurements in one experiment^6^. In this context, the use of automated robotic platforms is extremely useful since it ensures a high degree of technical reproducibility within and between experiments and keeps potential operator-induced bias to a minimum.

The overall goal of this HTS-HCI study was to provide a large set of high quality *in vitro* toxicity data for the purpose of an initial hazard-based ranking of MNMs in order to identify candidates for subsequent more detailed toxicological assessment. An additional aim was to use the dataset to explore the development of MNM-specific Quantitative Property Activity Relationships (QPARs) by combining the toxicity data with the physiochemical properties of the MNMs. To our knowledge, this database is the largest of its kind that covers such a wide selection of MNMs.

The creation of the database was initiated as part of the European Union funded project NanoMILE^7^, with resulting Knowledge Base^8^. Within the project a 3-tiered approach was adopted in which a preliminary series of MNMs was sourced from commercial suppliers and existing libraries such as the JRC nanomaterials repository (Batch 1). In parallel with this a second tier of in-house synthesised MNMs with known or predictable properties were prepared (Batch 2). Finally, as the project progressed and new needs were identified a third tier (Batch 3) of novel or highly tailored MNMs was developed on request to tackle specific problems or to test hypothesis identified during the testing of the first two batches^9^.

The MNMs were tested on the human hepatoma cell line, HepaRG. These cells show hepatocyte-like functions and express drug detoxifying enzymes at near *in vivo* levels^10^. This cell line has also been demonstrated to be suitable for use in HTS and HCI assays with chemicals^11^.

A total of 89 MNMs were tested which had different composition, size and surface properties, there were also present 3 coatings, 4 supernatants and 7 solvents/diluents. In all, 14 imaging endpoints were measured in the 15 experiments conducted. MNMs were tested at 10 different concentrations in 3 independent experiments (biological replicates). Four MNMs were tested in only two biological replicate-experiments because of their late delivery. Two other MNMs (PS-NH_2_ 50 nm and PS-COOH 100 nm) have no biological replicates.

**Fig. 1:**
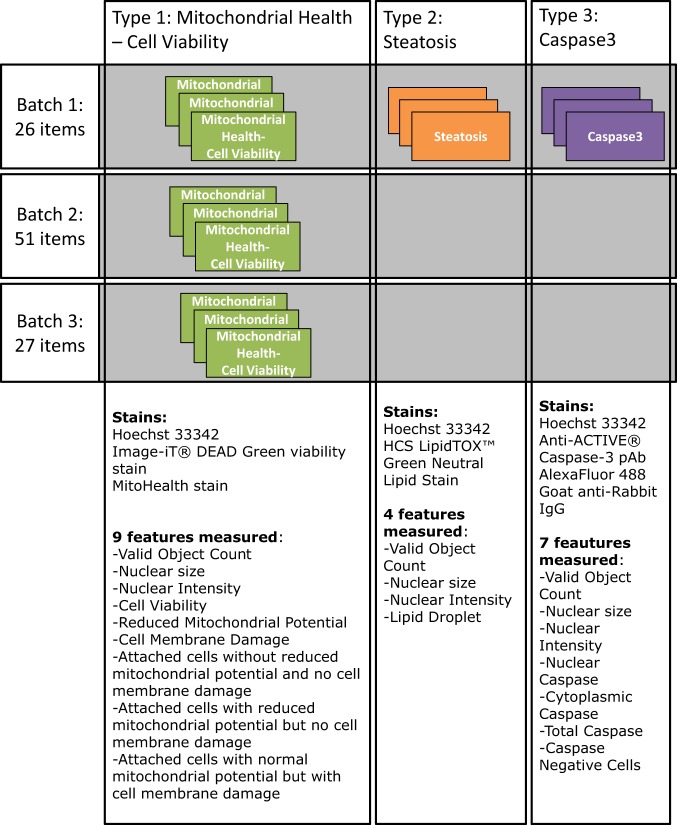
Graphical overview of the experiments performed.

A schematic overview of the experiments is provided in Fig. 1. The main type of experiment (type 1) addressed mitochondrial health and cell viability and was performed on all MNMs. This combined three different fluorescent dyes to analyse the nuclei, the state of the cell membrane (cell membrane damage), and the mitochondrial membrane potential. The HCI allows for a population analysis dividing the cell population into subpopulations with different characteristics defining the health of cells. For Batch 1, two additional types of experiments were performed, namely, detection of steatosis based on the detection of intracellular accumulation of neutral lipids (type 2), and measurement of apoptosis (type 3). All experiments included an analysis of cell nuclei.

## Methods

### Cell lines

The HepaRG human hepatic cell line was established by the INSERM (National Institute of Health and Medical Research) laboratory at Rennes, France. For this study, undifferentiated HepaRG cells were provided by Biopredic International (Rennes, France) in cryopreserved vials. The cells were maintained in culture medium consisting of William’s MediumE (Thermo Fisher Scientific, Melegnano, Italy) with 10% FBS (HyClone Fetal- Clone III, HyClone), 1% l-glutamine, 1% penicillin/streptomycin, 5 μg/ml bovine insulin and 50 μM hydrocortisone (all from Sigma, Milan, Italy). The cells were seeded at a density of 1 × 10^6^ cells into 75 cm^2^ flasks and the medium was refreshed every 2 to 3 days. Upon reaching confluence, the cells were transferred into 150 cm^2^ flasks. After 14 days, the cells start to differentiate into hepatocyte- and biliary-like cells. This process is supported by changing to differentiation medium, which consists of maintenance medium plus 1.7% DMSO (starting with 0.85% DMSO for 1 day). After 2 weeks in differentiation medium, the hepatocytes were selectively trypsinised and detached whereas the biliary cells remained adhered to the plate. The hepatocytes were then transferred into clear bottom black polystyrene 96-well microplates (5 × 10^4^ cells/well in 100 μL) by an automated procedure.

### Nanomaterials and chemical controls

As described above, 3 separate batches of test items were tested in a series of 15 HTS-HCI experiments. The Batch 1 contained 26 test items, consisting of 25 MNMs and 1 diluent. The Batch 2 contained 51 test items, of which 50 were mainly ultra-small MNMs and 1 was a supernatant. The Batch 3 contained 28 test items, consisting of 16 MNMs, 3 coating materials, 3 supernatants and 6 diluents. A reference MNM (PS-NH_2_) was included in all batches. Each of the experiments also contained HepaRG samples treated with chemical compounds with known effect which were used as controls to assess the performance of the robotic platform and of the staining. Type 1 experiments for mitochondrial health and cell viability contained Valinomycin and Cadmium Chloride. Type 2 experiments for steatosis contained CyclosporineA and type 3 experiments for apoptosis (Caspase 3) contained Cyclohexamide.

Overall the database includes data on 29 cerium dioxides, 17 silica, 12 titanium dioxides, 8 zinc oxides, 8 silver, 5 gold, 3 polystyrene, 3 calcium, 2 copper oxide, 1 barium, and 1 iron oxide MNMs with different sizes and coatings.

The reference MNM, PS-NH_2_, was selected for its known chemical and physical properties and behaviour *in vivo*^12,13^. A detailed discussion on the sequence of events leading to PS-NH_2_-induced death of astrocytoma cells can be found in the literature^13^. The sequence is as follows: increase of lysosomal volume – increased ROS levels – loss of lysosomal integrity – shrinkage of cell size – loss of mitochondrial membrane potential – loss of plasma membrane integrity – cell death. The impairment of mitochondria marks the point of no return for cell death. It has further been shown that the biomolecular corona of the PS-NH_2_ nanomaterial is retained during the uptake into the cell until they reach the lysosomes where the corona is degraded^14^, more specifically it was reported on different tested cell lines (HEK293, HepG2, hCMEC/D3, A549, SHSY5Y, 1321N1, RAW264.7)^15^.

The negative controls used were cells treated with medium. Additionally, cells were tested when exposed to water during the experimental design phase but no observable influence was detected. Within the experiment, the different diluents used for the MNMs were tested and are included in the DB.

In-depth characterisation of the tested MNMs was performed within the NanoMILE project and results are included in the excel sheet MNM characterisation which is part of the investigation file. Some additional characterisation of certain MNMs was carried out in other contexts, including for the functionalized SiO_2_ MNMs^16^. For the JRC TiO_2_ materials NM-103 and NM-104 the physico-chemical characterisation data are available on the JRC Nanomaterials Repository^17^ and are included in the report^18^. In the field of nano-bioscience, reporting standards have been developed to enhance the quality and reuse of published research^19^. As proposed therein, we provide information on size, shape, aggregation, zeta potential and density (mass/volume) of the majority of materials within the associated Metadata Record. Hence, if required for example, one could apply mathematical models^20^ to estimate the actual cellular dose.

Common protocols for particle dispersion and cell exposure, developed within the NanoMILE project, were used for all MNMs^21^. A summary of these dispersion protocols is included also in the JRC data catalogue^22^ and the characterisation file contains specific information of the applied protocol per MNM.

It is to be noted that all dispersions concerning ion releasing particles were always prepared at the end of each batch and immediately exposed to the cells in order to reduce the amount of free ions at the outset of the exposure. In general, the time between MNM dispersion and exposure varied from 30 to maximum 60 minutes.

### Cell treatment and staining

All the procedures for cell seeding, serial dilution of test items, cell treatment and cell staining were fully automated on Hamilton Star and Starlet platforms (Hamilton Italia Srl, Agrate Brianza, Italy). These robotic liquid handlers are equipped with 96-tip heads and installed either in a laminar hood (cell seeding, serial dilution of test items, cell treatment) or in a chemical fume hood (cell staining).

**Fig. 2:**
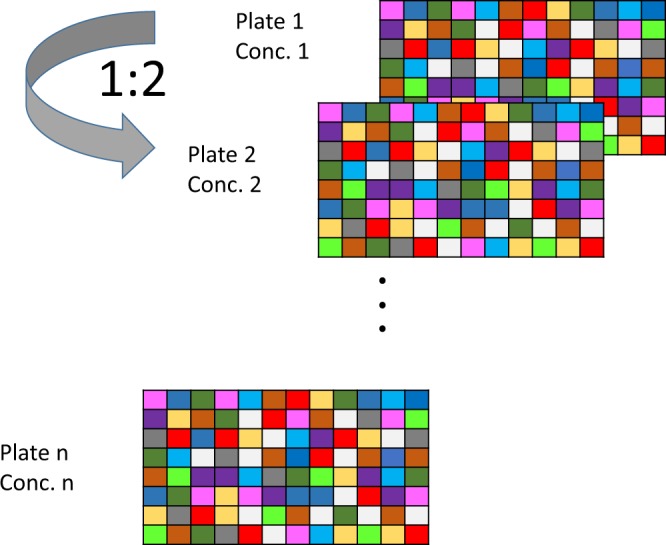
Graphical overview of the qHTS layout.

Plates were treated following a ‘quantitative HTS’ (qHTS) format, where all plates had the same plate layout and every plate corresponded to one tested concentration^23^. Thus for each experiment, 10 seeded 96-well-plates were used for the 10 concentrations tested. The highest particle concentration used was 250 μg/ml; followed by, plate by plate, serial 1:2 dilutions, the lowest concentration being 0.49 μg/ml (see Fig. 2).

**Fig. 3:**
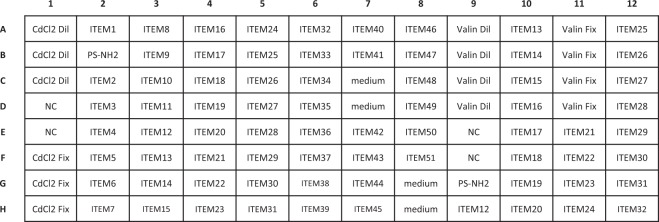
Example of the plate layout of run 2 of the type 1 experiment for the Batch 2 items (experiment 11). Here only ITEM12 to ITEM32 have technical replicates on the plate. For runs 1 and 3 (i.e. biological replicates) of the same type 1 experiment, the wells in columns 9 to 12 were treated with other ITEMS to have at least one technical replicate per ITEM. CaCl_2_ and Valinomycin were added as reference chemicals and are both diluted from starting concentrations of 11 μM and 165 nM respectively; i.e. are diluted following the 1:2 scheme like the MNMs or added manually at a fixed concentration on each plate.

For the experiments all 96 wells of the plates were used. The plate layout slightly changed from one experiment to another but followed a general concept. Namely, in addition to the tested items, each plate contained at least four negative control wells where cells were treated only with medium, and a number of wells with chemical controls. When possible, each plate contained two technical replicates of the tested items. For Batch 1 and Batch 3, all the tested items were present in duplicates on the plate but for the Batch 2 the size of the plate did not allow accommodating all 51 tested items in duplicates on a plate. Therefore, on each experiment the plate layout was modified in such a way that for every tested item two technical replicates were available in at least one of the three repeated experiments, i.e. biological replicates (see Fig. 3).

Cells were exposed to the test items for duration of 24 hours after which the cells were stained according to the experiment performed.

The main type 1 experiment (mitochondrial health and cell viability), was performed on all tested items and combined three different fluorescent dyes to analyse the nucleus (Hoechst 33342), the state of the cell membrane (cell membrane damage, Image-iT® DEAD Green viability stain), and the mitochondrial membrane potential (MitoHealth stain). These dyes are included in the HCS Mitochondrial Health Kit, Invitrogen Catalog no. H10295.

The type 2 experiment contained in addition to the nucleus staining dye (Hoechst 33342), also a dye for intracellular accumulation of neutral lipids (HCS LipidTOX™ Green Neutral Lipid Stain, Thermofisher Catalog no. H34475) and the type 3 experiment contained a dye for the detection of apoptosis (immunofluorescent staining using as primary antibody Anti-ACTIVE® Caspase-3 pAb, Promega Catalog no. G7481 and secondary antibody AlexaFluor 488 Goat anti-Rabbit IgG, catalog no. Abcam Ab150077).

The staining was performed on a separate Hamilton Starlet platform under a chemical safety hood dedicated to this purpose. Staining procedures applied are those provided by the kit producer. All the steps during staining were fully automated; the washing steps were performed by an incorporated automated washer with a 96-needle head for aspiration and for addition of wash solutions (typically PBS). The plates were processed in the same order as they were exposed to the nanomaterials (from C10, lowest concentration, to C1, highest concentration) and in case of need for medium removal, this was done using the robot’s 96-pipette head. More details on the processes and the preparation of solutions can be found in DB-ALM Method summary 167 (https://ecvam-dbalm.jrc.ec.europa.eu/).

### Automated cell image acquisition and data production

To acquire and analyse the fluorescence images of stained cells in the 96-well plates a Cellomics ArrayScan® VTI high content imaging instrument (Thermo Scientific) was used. This instrument is designed for high-capacity automated fluorescence imaging and quantitative analysis of fixed and living cells. It is composed of an inverted fluorescence microscope Zeiss™ Axio Z1 Observer from Carl Zeiss, a hotel which accommodates up to 45 standard 96-well-plates and a Catalyst Express robotic arm that feeds plates from the hotel into the microscope stage and back. The fluorescence microscope is equipped with motor driven stage, objective changer, autofocus system, and filter-wheel with different sets of filters and dichroic mirrors for fluorescence channel selection. The excitation light is delivered from a high intensity mercury vapour arc light source through a waveguide and the fluorescence images are recorded by a high sensitivity CCD camera.

The plate handling workflow is controlled with the scheduling software Polara and the image acquisition, analysis, visualisation, and storage with the HCS Studio™ Cell Analysis software (Thermo Scientific™ HCS Studio™ 2.0 Cell Analysis Software, Thermofisher Catalog no.: SX000041A). The HCS Studio™ Cell Analysis software is used for creating and modifying Assay Protocols. In Assay Protocols, users configure the acquisition parameters (e.g. objective, image resolution, number of fluorescence channels and their excitation/emission wavelengths, exposure times, number and position of images per well and wells per plate to be scanned), the assay parameters (type of image processing, object identification, selection, validation and analysis), and the cell and well features to be stored. The image analysis can be done either on-the-fly or after the acquisition. In this study the image acquisition and analysis were done separately as the on-the-fly approach slowed down the acquisition and sometimes overloaded the control computer due to the highly demanding calculations for image analysis. This approach was also used to iteratively reanalyse acquired images which was particularly useful for classification of the cells into the subpopulations; all properties are summarized in Online-only Table 1. The thresholds for cell classification were set based on cellular features calculated in the first image analysis, and subsequently applied in the second image analysis. To configure the Assays Protocols two Bioapplications were used; Compartmental Analysis.v.4 for type 1 and type 2 experiments and Colocalisation.v.4 for type 3 experiment. The objective 20x was used and 10 connected images in the middle of each well were recorded. The images from all experiments (432,000 images from 15 experiments * 96 wells * 10 concentrations * 10 fields_of_view * 3 channels) are stored and can be provided upon request via the JRC data catalogue^22^.

In all three types of experiments the cellular nuclei were stained with the Hoechst 33342 dye and imaged in the channel 1. This stain provided a marker for the cell identification and their total counts ‘Valid Object Count’, the average ‘Nuclear size’ and the average ‘Nuclear Intensity’ were calculated across all the cells in a well and stored in the database.

In the type 1 experiment, the ImageDeadGreen dye stained the cells which had cell membrane damage and these were imaged in channel 2. The average pixel intensity of this stain in the area of nucleus for each identified cell was calculated and averaged across all the cells in a well and then used as an indicator of the ‘Cell Membrane Damage’. In channel 3 the MitoHealth dye which stained the cytoplasmatic mitochondria was imaged and then used as an indicator of its mitochondrial membrane potential. The cytoplasm was defined as a ring of a predefined size around the selected nuclei and the average pixel intensity was calculated and averaged across all the cells in a well and then used as an indicator of the ‘Reduced Mitochondrial Potential’. Additionally, the cells were categorised into four phenotypic sub-populations. The combination of two selection criteria was used: normal or reduced mitochondrial potential, and normal or damaged cell membrane. The portion of the cells in three of these four phenotypic sub-populations are in the database: ‘Attached cells without reduced mitochondrial potential and no cell membrane damage’, ‘Attached cells with reduced mitochondrial potential but without cell membrane damage’, and ‘Attached cells with normal mitochondrial potential but with cell membrane damage’ whereas the fourth sub-populations can be calculated as what remains to arrive at 100%.

In the type 2 experiment, the steatotic cells were identified by imaging the accumulated lipid droplets in their cytosol which were stained with the HCS LipidTOX™ Green Neutral Lipid Stains. The total area of the lipid droplets in each cell was calculated and then averaged across all the cells in a well and used as the marker for steatosis ‘Lipid Droplet’.

In the type 3 experiment, the apoptotic cells were identified by using the immunostaining Anti-ACTIVE® Caspase-3 pAb - AlexaFluor 488 Goat anti-Rabbit IgG. In each cell the presence of this stain for cleaved Caspase-3 was quantified separately in the nucleus and cytosols. The cells were then categorised into three phenotypic sub-populations, having either no Caspase-3 ‘Caspase Negative Cells’, Caspase-3 only in the cytoplasm ‘Cytoplasmic Caspase’ for detection early apoptosis, or in the nucleus or both the cytosol and the nucleus ‘Nuclear Caspase’ for late apoptosis. On top of that the overall mean Caspase-3 staining ‘Total Caspase’ was calculated over all cells in a well.

### Normalization of the data

The raw data files per plate were loaded into Excel and merged to produce a single data file per experiment. As a first step, the data were analysed experiment by experiment following the signal-to-noise ratio approach^24,25^. The following steps were followed separately for every feature (endpoint) within one experiment:

- Step 1: On each of the plates (labelled by subscript i) the average response of the 4 negative controls was calculated for each feature (µ_ci−_), to obtain the average plate behaviour without MNM influence.
- Step 2: The difference between a negative control signal and the average negative control signal was defined as noise. So, for each plate, 4 noise values were obtained by subtracting the individual negative control responses from their plate average.
- Step 3: The noise data from the separate plates was put together to get, and aggregated to an overall standard deviation of the noise signal for the experiment (σ_noise_). It was obtained using the 4 ‘noise signals’ per plate from all plates, so a total of 40 noise signals were used to calculate an overall standard deviation. This ensured that the overall standard deviation was calculated from a large enough set of data. The negative control signal on each plate and the overall standard deviation were used to normalize the data for each of the test items on the plate. This measure of standard deviation is used to quantify the amount of variation or dispersion of a set of data values.
- Step 4: If available, two technical replicates of the feature for each concentration were averaged for each tested item, otherwise the individual value for the item concentration was used (µ_xi_).
- Step 5: The plate average negative response (step 1) was subtracted from the average item signal (step 4). This way a pure item response was obtained as any potential plate-specific effects were removed.
- Step 6: The difference from step 5 was then compared to the overall noise signal by dividing:

$${z}_{xi}=\left({\mu }_{xi}\,-\,{\mu }_{ci-}\right)/{\sigma }_{noise}$$

For each item-feature combination within an experiment, using the information of all 10 plates, a dose response curve was obtained using the signal-to-noise data.

This normalization allows comparing data from different features and experiments on one plot and immediately provides an idea if the effect induced by a test item is of significance using, for example, a threshold of −3 (downwards response) or 3 (upwards response) which corresponds to a 99% certainty that the cell behaviour is different from the control value under the assumption that the difference between the two signals is normally distributed.

The feature results of the same item tested at a certain concentration can also be combined across experiments by averaging z_xi_. For the creation of the presented database every item-feature combination was tested for all concentrations in three independent experiments. The average of three different experiments is presented in the database. As is the case for a single experiment, assessment of the significance of the signal can be done by comparing the average of the three experiments to the thresholds −3/√3 and 3/√3 (with 99% certainty).

## Data Records

All data is available at the JRC data catalogue^22^. A general Excel file named ‘Investigation’ contains an overview of the individual experiments, the name of files with the raw data (also included as Table 1), and the metadata of all assays. The file is complemented by a separate sheet containing all the available characterisation data of the nanomaterials labelled ‘DB characterisation’. This sheet also contains the information on the dispersion protocol used and the availability of the nanomaterials themselves. For each experiment, the raw data files are within the subfolder\RawData. There is one Excel file per experiment containing plate per plate (stored on separate sheets) recorded data. Sheet ‘plate 1’ refers to the all relevant endpoints from the highest concentration plate while sheet ‘plate 10’ refers to the lowest concentration plate. The plate layouts of all experiments are also included in the ‘Investigation’ Excel file.

**Table 1:** Experimental study raw data sources.

Experiment number	Date	Type of experiment	Timepoint	Filename
1	13/02/2014	Steatosis	24 h	Experiment1-Batch1-Steatosis.xlsx
2	17/02/2014	Caspase3	24 h	Experiment2-Batch1-Caspase3.xlsx
3	24/02/2014	Steatosis	24 h	Experiment3-Batch1-Steatosis.xlsx
4	27/02/2014	MH-CellViability	24 h	Experiment4-Batch1-MitoHealth-CellViability.xlsx
5	27/03/2014	MH-CellViability	24 h	Experiment5-Batch1-MitoHealth-CellViability.xlsx
6	24/11/2014	MH-CellViability	24 h	Experiment6-Batch1-MitoHealth-CellViability.xlsx
7	27/11/2014	Steatosis	24 h	Experiment7-Batch1.Steatosis.xlsx
8	01/12/2014	Caspase3	24 h	Experiment8-Batch1-Caspase3.xlsx
9	15/12/2014	Caspase3	24 h	Experiment9-Batch1-Caspase3.xlsx
10	03/08/2015	MH-CellViability	24 h	Experiment10-Batch2-MitoHealth-CellViability.xlsx
11	30/11/2015	MH-CellViability	24 h	Experiment11-Batch2-MitoHealth-CellViability.xlsx
12	14/12/2015	MH-CellViability	24 h	Experiment12-Batch2-MitoHealth-CellViability.xlsx
13	13/06/2016	MH-CellViability	24 h	Experiment13-Batch3-MitoHealth-CellViability.xlsx
14	20/06/2016	MH-CellViability	24 h	Experiment14-Batch3-MitoHealth-CellViability.xlsx
15	27/06/2016	MH-CellViability	24 h	Experiment15-Batch3-MitoHealth-CellViability.xlsx

A subfolder\TransformedData contains files with the transformed data for each of the experiments. In each file the data from all plates are grouped together on one sheet per endpoint. In each of these sheets, the first set of columns labelled ‘Original Data’ contains the averaged raw data of the technical replicates (on plate) while the second set of columns labelled ‘Transformed Data’ contains the signal-to-noise transformed data per MNMs.

Averaging the transformed data across the three experiments, which represent three biological replicates, lead to the values recorded in the final DB for each NMMs-endpoints-concentration combination.

In addition, a subfolder\Methods contains a document with details on the three protocol versions for dispersion of MNMs (depending on their hydrophobicity) and the three source codes for the data acquisition.

Physiochemical properties of the MNMs are stored in a separate sheet from the toxicity results obtained within this experiment. Each MNM has a unique name and identifier so merging the sheets to querying toxicity data based on physiochemical properties is possible.

## Technical Validation

For the technical validation of the data a stepwise approach was followed. First the data within an experiment was validated, followed by a validation across experiments within one type of experiment. Finally, data across experiment types were compared.

### Stability of each individual experiment

The data from all control wells of the different plates within an experiment were put next to each other graphically. Confirmation on the stability was checked in various ways.

It was checked if the negative controls within a plate and within the experiment were stable. An example from the experiment 10 is shown in the Table 2. The coefficient of variation within each plate varied from 3% to 15%. At the same time, the coefficient of variation obtained from all ten plate averages was less than 10%.

**Table 2:** Data from negative controls for experiment 10.

		Plate										Average across plate	SD across plates	CV of Plate average
		10	9	8	7	6	5	4	3	2	1			
Raw Signal	NC-1	479	490	526	557	630	514	535	584	607	628			
	NC-2	504	511	517	573	593	559	581	548	583	656			
	NC-3	471	476	506	480	487	539	508	516	535	630			
	NC-4	524	506	614	593	628	554	548	628	548	701			
Plate average		495	496	541	551	584	541	543	569	568	654	554	46	8%
Plate SD		24	16	49	49	67	20	30	48	33	34			
Plate CV (%)		5%	3%	9%	9%	12%	4%	6%	8%	6%	5%			

A similar stability check was performed on the wells with the positive control chemicals. Table 3 shows that the signal in the wells where the positive control chemicals where at fixed concentrations across the plates, were stable within an experiment. The coefficient of variation for the three added chemicals was between 6 and 14%.

**Table 3:** Data from Positive control for experiment 10.

		Plate										Average across plate	SD across plates	CV	z’-factor using plate averages
		10	9	8	7	6	5	4	3	2	1				
VAL 165 nM-Fix	Raw Signal	196	208	207	225	210	213	222	236	233	239				
		170	197	185	215	229	219	223	232	202	268				
	Plate average	183	202	196	220	219	216	223	234	217	253	216	20	9%	0.42
VAL 7.2 µM -Fix	Raw Signal	151	176	176	188	193	187	184	248	184	188				
		144	171	168	186	175	179	184	255	184	214				
	Plate average	148	174	172	187	184	183	184	251	184	201	187	26	14%	0.41
CdCl2 100 µM-Fix	Raw Signal	162	167	159	174	158	147	157	156	152	164				
		163	148	154	165	158	136	142	140	164	163				
		155	163	151	180	178	122	170	174	147	163				
	Plate average	160	159	155	173	164	135	156	157	154	163	158	10	6%	0.58

**Fig. 4:**
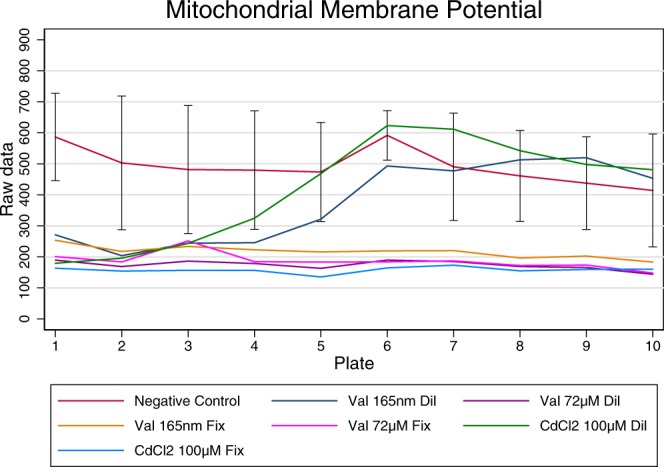
Behaviour of controls of the Mitochondrial Membrane Potential endpoint (experiment 10).

Additionally, Fig. 4 demonstrates that the positive controls induced an evident effect compared to the negative controls, i.e. a reduction of the signal in the case of the mitochondrial membrane potential. In order to confirm the quality of our assay we used the z’-factor where the mean and the standard deviation of the positive controls within a plate were compared with the mean and the standard deviation of the negative controls. A classic HTS format^26^ normally includes a large number of positive controls per plate. For this qHTS format, with small number of controls within a plate, we used the negative and positive control signals present on all plates at a fixed concentration to obtain a z’-factor for the experiment. The averages within a plate were used for the calculation of the z’-factor which was 0.58 for Cadmium Chloride at 100 µM, thus confirming the quality of our qHTS assay (see Table 3).

Besides the fact that the wells treated with the chemical controls at the high concentration showed their significant effect on the cells with respect to the negative control wells, it can also be observed that these compounds do show a classic Hill dose response behaviour when the compound is diluted across the plates (dilution factor 2). For the wells treated with the highest dose (present on plate 1) the signal is significantly different from that of the negative control, while for the wells where the lowest doses were applied (present on plate 10) the signal is similar to the negative control wells (See Fig. 4).

### Verification of responses of MNMs within an experiment

Overall, the performed experiments did not suffer from any special effects. Neighbouring wells did not influence each other nor did the experiments suffer from border effects. This was all verified up front by preliminary test experiments and no correlations between neighbouring items were observed. If effects were observed they always followed expected dose response relationships i.e. low dose caused low response and high dose caused high response, often having a Hill or exponential shape.

### Comparison of results between and within experiments

**Fig. 5:**
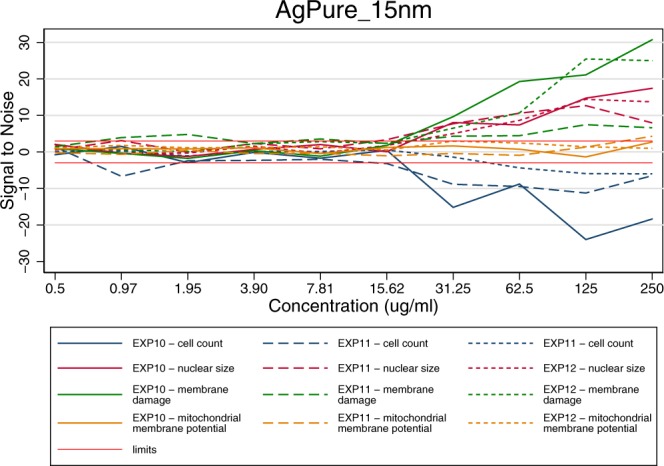
Comparison of results for AgPure (15 nm) between different endpoints for three different experiments of the mitochondrial health – cell viability experiment in Batch 2. The limits indicate the Signal to Noise reference lines at −3 and 3.

In order to have a clear idea of the bioactivity of the MNM in relation to different endpoints, the replicated measurements were analysed following the’signal-to-noise’ approach described above. The normalization allows the combination and comparison of the dose responses curves of different endpoints, different experiments, as well as different MNMs. Figure 5 shows an example of the induced effect on exposing HepaRG cells to different concentrations of AgPure (15 nm) for different features and for the 3 different experiments of the same type.

### Combining the different experiments

**Fig. 6:**
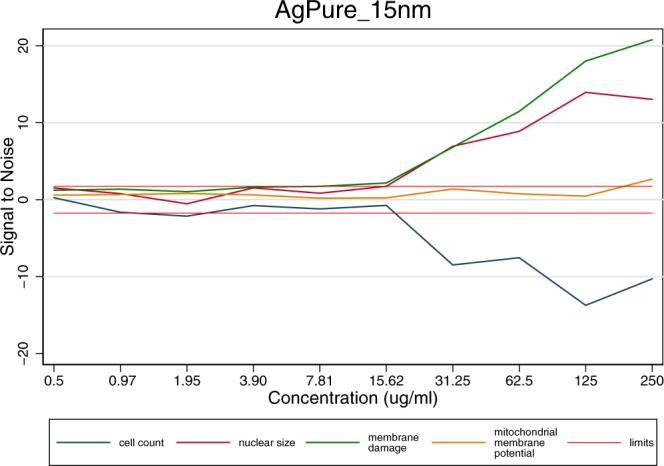
Results of averaged values per endpoint and concentration across three independent experiments for AgPure (15 nm). The limits indicate the Signal to Noise reference lines.

Given the reproducibility of the three biological replicates, data were averaged across experiments for each of the items-concentration and endpoint. Following this approach Fig. 5 was summarized to Fig. 6, leading also to the data available in the final database.

### Combining the data from different type of experiments and different batches

The raw data provides in total nine data sets for the tested items in Batch 1 (three for each type of experiment) and three data sets for Batch 2 and one for Batch 3.

The three types of experiments performed on Batch 1 all contain the Hoechst stain through which data is obtained for cell count, nuclear size, nuclear intensity. So for all 9 datasets those three endpoints can be used to analyse comparison. The same results in terms of the dose response curve shape and the point of departure were observed for all items, and therefore it was concluded that the data of different types of experiments of Batch 1 can be combined and complement each other. Although one could use the overall average of all 9 experiments for cell count, nuclear count and nuclear intensity, we opted to include within the database only the average of the three obtained in the type 1 experiment in order to be consistent with the remaining part of the database. Nevertheless, all raw and transformed data are available in the Excel files.

Finally, a consistency check between the different batches was also performed. The results of all features for the present controls (positive control chemicals and reference MNM) were compared. All dose response curves showed high consistency of behaviour.

As a final result a database was constructed. It contains for each MNM one reliable dose response curve per endpoint obtained as an average of three independent experiments using the ‘signal-to-noise’ approach. The database is visualized in a heatmap^27^ where each row represents a MNM or relevant tested item and the columns contain the different endpoints for increasing concentrations. The colour code used goes from dark red identifying a strong reduced ‘signal-to-noise’ to dark green for intermediate values moving then to dark blue identifying a strong increased ‘signal-to-noise’.

Materials are not grouped following a cluster analysis but according to their nano-form or relevance of nano-form. The upper part of the database includes the controls that were included in the various experiments. Besides MNMs in their pristine and aged form, the database also compares different coatings and sizes and, in some cases, also the supernatant is included to exclude specific effect of those.

### ISA-Tab metadata file

Download metadata file

## Data Availability

Codes used for image processing and data acquisition for the three types of experiments have been made publically available and are included in the JRC data catalogue^22^.

## Online-only Table

**Online-only Table 1:** Details on cellular measurements and classification of cells into sub-populations.

Assay name	Stain/Filter Set/Channel	BioApplication	Endpoint nomenclature used in DB	Feature name from Bioapplication	Definition of the feature	Biological meaning of the feature
Mitochondrial Health-Cell Viability	Hoechst 33342/XF93-Hoechst/Ch1 Image-iT® DEAD Green viability stain/XF93-Fitc/Ch2 MitoHealth stain/XF93-Tric/Ch3	Compartmental Analysis v.4	**Valid Object Count**	ValidObjectCount	Number of valid objects identified in the well (Ch1 object selection parameters)	Total cell count (number of nuclei)
			**Cell Viability**	not directly measured	ValidObjectCount x (%EventType1Objects + %EventType3Objects)	Living cell count (number of nuclei)
			**Reduced Mitochondrial Potential**	MEAN_RingAvgIntenCh3	Average of (Average intensity of all pixels within the ring mask) of all objects in the well	Average pixel intensity of the MitoHealth marker in cytoplasm (excl. nucleus) for all cells
			**Cell Membrane Damage**	MEAN_CircAvgIntenCh2	Average of (Average intensity of all pixels within the circmask) of all objects in the well	Average pixel intensity of the ImageDeadGreen dye in nucleus for all cells
			**Nuclear size**	MEAN_ObjectAreaCh1	Average of the area of all CH1 objects in the well	Average nuclear size
			**Nuclear Intensity**	MEAN_ObjectAvgIntenCh1	Average of (Average intensity of all pixels within Ch1 object) of all CH1 objects in the well	Average nuclear pixel intensity
			**Attached cells without reduced mitochondrial potential and no cell membrane damage**	%EventType3Objects	(NOT CircAvgIntenCh2) AND RingAvgIntenCh3	Fraction of cells with impermeable cell membrane and normal mitochondria membrane potential (in %)
			**Attached cells with reduced mitochondrial potential but without cell membrane damage**	%EventType1Objects	(NOT CircAvgIntenCh2) AND (NOT RingAvgIntenCh3)	Fraction of cells that have mitochondrial function altered, but are not dead yet (no DeadGreen, dark MitoHealth) (in %)
			**Attached cells with normal mitochondrial potential but with cell membrane damage**	%EventType2Objects	CircAvgIntenCh2 AND (NOT RingAvgIntenCh3)	Fraction of cells that have both the membrane permeable for dead cell marker (DEAD Green) and the decrease of mitochondria membrane potential (in %)
Steatosis	Hoechst 33342/XF93-Hoechst/Ch1 HCS LipidTOX™ Green Neutral Lipid Stains/XF93-Fitc/Ch2	Compartmental Analysis v.4	**Valid Object Count**	ValidObjectCount	Number of valid objects identified in the well (Ch1 object selection parameters)	Cell count (number of nuclei)
			**Nuclear size**	MEAN_ObjectAreaCh1	Average of the area of all CH1 objects in the well	Average nuclear size
			**Nuclear Intensity**	MEAN_ObjectAvgIntenCh1	Average of (Average intensity of all pixels within Ch1 object) of all CH1 objects in the well	Average nuclear pixel intensity
			**Lipid Droplet**	MEAN_RingSpotTotalAreaCh2	Average of (total area of all spot pixels within the ring mask) of all objects in the well	Average area of the acumulated neutral lipids in cytoplasm (excl. nucleus) per cell
Caspase3	Hoechst 33342/XF93-Hoechst/Ch1 Anti-ACTIVE® Caspase-3 pAb - AlexaFluor 488 Goat anti-Rabbit IgG/XF93-Fitc/Ch2	Colocalization v.4	**Valid Object Count**	ValidObjectCount	Number of valid objects identified in the well (Ch1 object selection parameters)	Cell count (number of nuclei)
			**Nuclear size**	MEAN_ObjectAreaCh1	Average of the area of all CH1 objects in the well	Average nuclear size
			**Nuclear Intensity**	MEAN_ObjectAvgIntenCh1	Average of (Average intensity of all pixels within Ch1 object) of all CH1 objects in the well	Average nuclear pixel intensity
			**Nuclear Caspase Cells**	%HIGH_ROI_A_Target_I_and_II_OverlapArea	Percentage of objects in the well with Feature: ROI_A_Target_I_and_II_OverlapArea above a certain limit	Fraction of cells with caspase 3 in either nucleus or in both, nucleus and cytoplasma, (in %)
			**Cytoplasmic Caspase Cells**	not directly measured	100-%HIGH_ROI_A_Target_I_and_II_OverlapArea-%LOW_ObjectCountCh2	Fraction of cells with caspase 3 in cytoplasma only (in %)
			**Total Caspase**	MEAN_ObjectTotalIntenCh2	Average of the total intensity of all pixels within modified channel 1 object mask in channel 2	Average total intensity of caspase 3 stained objects
			**Caspase Negative Cells**	%LOW_ObjectCountCh2	Percentage of objects in the well with Feature: ObjectCountCh2 below a certain limit	Fraction of cells with no caspase3 stained objects (in %)
